# Interdisciplinary Simulation Training Reduces Restraint Use in the Emergency Department: A Pilot Study

**DOI:** 10.7759/cureus.39847

**Published:** 2023-06-01

**Authors:** Gary Duncan, Brad Gable, Megan Schabbing

**Affiliations:** 1 Medical Education and Simulation, OhioHealth, Columbus, USA; 2 Emergency Medicine, OhioHealth, Columbus, USA; 3 Psychiatry and Behavioral Sciences, OhioHealth, Columbus, USA

**Keywords:** workplace violence, interdisciplinary teaching, interdisciplinary simulation, healthcare simulation, de-escalation, healthcare worker safety, patient safety and quality improvement

## Abstract

Introduction

Safe and effective management of agitated patients poses multiple challenges for healthcare professionals. Patients placed in restraints because of agitated behavior are at a higher risk of complications, including death. This intervention was designed to provide emergency department staff a framework for de-escalation, improve teamwork, and reduce the use of violent physical restraints.

Methods

Emergency medicine nurses, patient support associates, and protective services officers underwent a 90-minute educational intervention in 2017. A 30-minute lecture focusing on communication and early use of medication for agitation was followed by a simulation using standardized participants, then a structured debriefing. A standardized return-on-learning tool determined participants’ reactions to and application of the educational intervention. Additionally, data was collected and reported as a ratio of number of restraints applied each month compared to total emergency department visits that month. Data were analyzed comparing the six months before the education and the subsequent six months after the education.

Results

A pilot group of 30 emergency department staff members completed the educational intervention. The intervention contributed to the overall decrease in restraint use in the department. Most participants (86%) felt more confident in their ability to manage agitated patients.

Conclusion

An interdisciplinary simulation-enhanced educational intervention successfully reduced use of restraints in the emergency department and improved staff attitudes toward de-escalation techniques for agitated patients.

## Introduction

Workplace violence (WPV) is a well-known issue in the healthcare system, particularly in the emergency department (ED) [1]. Recent studies have shown an incidence of WPV of 47% for doctors, with up to 95% of nurses reporting verbal WPV within the past year [1-3]. Agitation and aggression among patients can lead to WPV faced by healthcare providers [1-3]. A recent study found that lack of violence management training was one of the leading causes of nurse dissatisfaction with the handling of WPV [2]. Understanding how to verbally de-escalate a patient who is becoming aggressive is an important first step in minimizing WPV related to patient agitation [1,4]. Trainings dedicated to simulated interdisciplinary de-escalation have demonstrated improvements in knowledge and confidence regarding de-escalation and show high satisfaction among nursing and security staff who have undergone training [5,6]. Simulated patient scenarios of aggression have high satisfaction scores among participants because standardized patients can increase the sense of realism [7]. 

Restraint use in the ED has been linked to increased morbidity and mortality in agitated ED patients [8,9]. Despite this risk to the patient, up to 84% of agitated patients in the ED are placed in restraints during their stay [10]. In addition, patients placed in restraints express feelings of loss of freedom and personal dignity [11]. The most common demographic groups to be restrained are bimodal distributions of young males with alcohol and drug use and elderly adults with medical comorbidities [12]. Therefore, early identification of these patients may be beneficial in reducing restraint use [13]. Multiple studies have shown that verbal de-escalation and use of oral medications are safer for the patients and staff, and initiating these measures is an important first step in the management of an agitated patient [1,4,14].

In this study, we sought to increase confidence and knowledge of staff de-escalation techniques through a combined didactic and simulated patient encounter. Nurses, protective services officers, and Patient Support Associates (PSAs) were included in the course to improve interdisciplinary communication and knowledge, which has been shown to be beneficial in de-escalation preparedness [5]. Simulated patient encounters with dedicated teaching have the primary aim of increasing staff confidence in management of an agitated patient, which is an important first step for improving the safety of patients and staff [1,7,14-16]. A secondary measure from this pilot study is the change in restraint use by the department in the six months after undergoing training to determine whether a focus on education in verbal de-escalation has an effect on restraint use in the ED [17].

## Materials and methods

Study design and setting

The psychiatric emergency unit of our large 88,000-visit-per-year community-based academic hospital’s Emergency Department was identified as having a relatively higher volume of agitated patients and staff assaults. A group of content experts from psychiatric emergency services and simulation education specialists developed the curricular educational content, goals, and objectives. Since the experience of the learners with verbal de-escalation techniques varied, we sought to follow the revised Bloom’s taxonomy for providing education [18]. With that principle in mind, the session progressed from remembering and understanding concepts of de-escalation via a didactic session with facilitated discussion, to application and evaluation of the knowledge and skills obtained by the learners through a simulated patient encounter and debriefing. 

Selection of participants

Learners were identified as a purposive sample of nurses, PSAs, and protective services officers from the emergency department who were available to attend the education sessions. Leadership identified the emergency department nurses and PSAs who most commonly worked in the psychiatric emergency unit of the emergency department. Sessions were offered on multiple days at variable times to increase participation of staff members from all shifts.

Intervention

A 90-minute simulation-based educational intervention was determined to be the most appropriate method of education by expert consensus. The intervention began with a short introduction in the form of a pre-briefing in which expectations were clarified and psychological safety was emphasized [19]. Next, learners were engaged in a 30-minute didactic lecture and discussion led by a psychiatric emergency medicine expert. The objectives for the didactic session were as follows: (1) create a culture of dignity and respect for the patient; (2) show attentiveness to the emotional safety of the patient; (3) develop a framework to empower each staff member to optimally manage a behavioral health emergency; and (4) understand each team member’s role as part of the team when managing a behavioral health emergency. Immediately following the didactic education, learners participated in a simulated case involving an agitated patient. The case was based on actual clinical scenarios in which agitated patients had presented to the psychiatric emergency unit. In this simulation, the standardized patient portrayed a schizophrenic patient who had recently stopped taking his/her psychiatric medications and subsequently experienced paranoid delusions. Ideal management by learners would result in the patient de-escalating. For learners who were unable to de-escalate the patient, the case was terminated prior to when patient would have been placed in violent physical restraints. Most simulation sessions took approximately 10-15 minutes to complete. This methodology has been previously described for patients in a different hospital setting by this research team [20]. Following the simulation, expert debriefers facilitated a 45-minute debriefing session. Not only did learners reflect on the de-escalation techniques they had employed, but they also provided insights into potential improvements in teamwork and communication that were then communicated to department leadership as feedback.

Learners participated in the 90-minute simulation-based educational intervention in groups of four. Each learner group consisted of two nurses, one PSA, and one protective services officer. The learner groups were designed with this configuration to best approximate the staffing of the psychiatric emergency unit on any given day. A total of 10 education sessions were offered, and some officers participated twice (although they only completed the post-education survey once). The educational sessions were conducted over two days at the end of June 2017.

This project was reviewed by the OhioHealth Institutional Review Board and did not meet criteria for human subjects research but was considered a quality improvement project.

Measurements

We evaluated our educational intervention using our standardized Return on Investment in Learning (ROL) methodology that is based on the Phillips Return on Investment (ROI) methodology [21]. ROL levels zero, one, two, and four (of a possible five) were evaluated in this study. Level zero counted the participants; level one evaluated learner reactions to the education; level two evaluated knowledge gained; level four evaluated patient impact of the education. Learners were provided a survey immediately after the 90-minute simulation-based educational intervention. This Likert-based scale measures the impact of the training. These survey questions evaluated (1) self-perceived attitudes and confidence, (2) relevance to clinical practice, and (3) likelihood of applying the knowledge. In addition, we were able to extract restraint data from our electronic medical record. Specifically, the number of violent restraints applied was able to be determined on a monthly basis. In addition, we determined the total number of emergency department visits per month.

Survey scores were evaluated using the RedCap survey program. Statistical analyses were performed using IBM SPSS Statistics version 25.0 (Armonk, NY, USA) based on traditional two-sided tests with the alpha error set at 5%. Likert scale responses were reported as mean values. In addition, qualitative learner feedback was also captured in the survey and reported in the discussion.

## Results

A total of 30 discreet learners participated in the training including nurses (10), PSAs (9), and protective services officers (10) (one participant did not disclose his or her profession, and did not complete the survey). At the time, there were 115 full and part-time nurses employed in the emergency department. This intervention trained 8.7% of the nursing staff. As a pilot study, these learners were asked to evaluate the simulation-based educational intervention.

An overwhelming majority of participants either agreed or strongly agreed that the training was relevant to their work (86.2%). Another 89.7% of participants responded that the training provided them with new or clarified existing information, and 93.1% responded that they intended to use what they learned from the training in future practice.

Some positive qualitative feedback received included that the training was “relevant to my work when all on the same page” when managing an agitated patient. Another learner spoke about the importance of the training and stated that we “as caregivers need to check our egos at the door” when involved in these situations. Although a substantial majority of protective services officers found the training beneficial, they were the most likely individuals to feel that the content was not relevant to their work with qualitative statements from officers reporting that the training is “very hard to apply in the protective services role” and one who felt that “Protective services doesn’t get a lot from this.”

The four key learning objectives stated above were met by over 86% of participants in all categories (Table 1). Participants agreed or strongly agreed that they felt more confident in their ability to: provide a culture of respect and dignity for the patient (89.7%), provide emotional safety for agitated patients (89.7%), use a framework to approach agitated patients (86.2%), and identify each team member’s role within an interdisciplinary approach to agitated patients (86.2%). One hundred percent of learners felt that the instructors were knowledgeable about the subject matter and 72.4% of participants (21/29) felt that the course was “very good,” the highest rating scored. To that end, 89.7% of participants felt that the training would be beneficial to their colleagues.

**Table 1:** Return on Learning Assessment

No.	Question	Number that agree or strongly agree (strongly agree)	Percent that agree or strongly agree
2.	I feel more confident in my ability to provide a culture of respect and dignity for the patient	26/29 (8)	89.7%
3.	I feel more confident in my ability to provide emotional safety for agitated patients	26/29 (9)	89.7%
4.	I feel more confident in my ability to use a framework to approach agitated patients	25/29 (8)	86.2%
5.	I feel more confident with my role within the interdisciplinary approach to agitated patients	25/29 (6)	86.2%
6.	I feel more confident in understanding other team members’ roles in the interdisciplinary team for agitated patients	26/29 (10)	89.7%
7.	I feel more confident with my ability to safely manage agitated patients	24/29 (9)	82.8%
8.	This training was relevant to my work	25/29 (16)	86.2%
9.	This training provided me with new information (or clarified old information)	26/29 (15)	89.7%
10.	I intend to use what I learned from this training	27/29 (17)	93.1%
11.	This training would be of benefit to my colleagues	26/29 (21)	89.7%
12.	Overall, I thought the training was beneficial	28/29 (21)	96.5%
13.	I thought the instructor(s) were effective	29/29 (24)	100%

**Figure 1:**
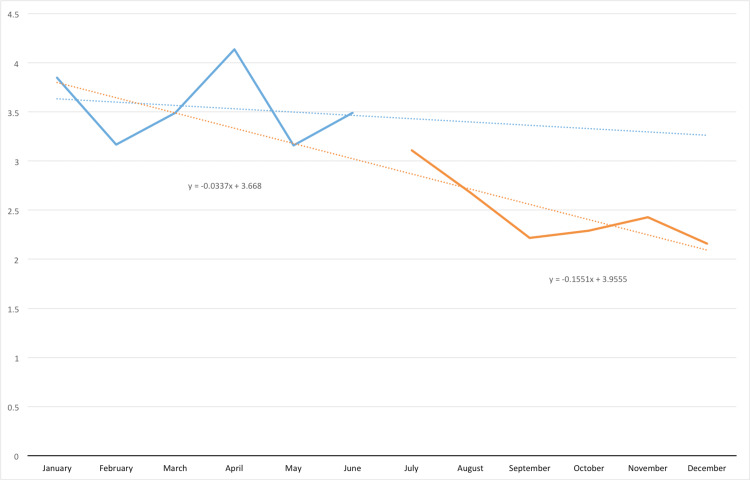
Emergency Department Violent Physical Restraint Use Rate (Number of Restraints/1000 Visits) 2017

Regarding restraint use in the emergency department, over the six months prior to the course, January 2017 through June 2017, the rate of restraint use for violent patients in the emergency room averaged 3.55 (95% CI 3.242 - 3.858) incidences of violent restraints per 1000 ER visits. In the six months after intervention, July 2017 through December 2017, that rate reduced to 2.48 (95% CI 2.192 - 2.768) incidences of restraint per 1000 ER visits which is a statistically significant decrease (p=0.00055) (Tables 2, 3). The slope of change during the six-month period after intervention was -0.155 which is a change from the six months prior slope of -0.0337 (Figure 1).

**Table 2:** Violent Physical Restraint Use in the Emergency Department

Month	Number of Restraints Used	Total Patient Visits	Violent Physical Restraint Use Rate per 1000 visits
January 2017	32	8306	3.85
February 2017	24	7576	3.17
March 2017	29	8316	3.49
April 2017	33	7975	4.14
May 2017	26	8223	3.16
June 2017	27	7743	3.49
July 2017	26	8366	3.11
August 2017	22	8218	2.68
September 2017	18	8100	2.22
October 2017	18	7885	2.29
November 2017	18	7399	2.43
December 2017	16	7407	2.16

**Table 3:** Two-Tailed T-Test Analysis of Violent Physical Restraint Use in the Emergency Department 2 tailed T-test = 4.9680; p-value = 0.0006

	N	Mean	S2 (population variance)	S (standard deviation)	95% Confidence Interval
Pre-Intervention (Jan 2017- Jun 2017)	6	3.55	0.14836	0.385	3.55 +/- 0.308 (3.242 - 3.858)
Post-Intervention (Jul 2017- Dec 2017)	6	2.48	0.129	0.359	2.48 +/- 0.288 (2.192 - 2.768)

## Discussion

Through the didactic session and simulated patient encounter, this simulation-based educational intervention was able to significantly reduce the number of times patients were restrained due to violence in the emergency room over a six-month period. This effect was achieved even with only a small portion (10 of 115 full and part-time nurses) of ED staff undergoing the training. Restraint use has been associated with detrimental mental and physical consequences to the patient, both in the short-term and long-term [10]. Patients have stated that restraints made them feel as if they were being treated like animals and reported having sustained injuries during the restraining process that developed into long-term pain [9-10]. By reducing restraint use in the emergency department, this simulation-based education will hopefully provide a more patient-centered approach to agitated patients throughout the hospital. Previous studies regarding verbal de-escalation training have focused on initial reactions to training [1-2,7,20]. Not only does our study show a positive initial response to the education, but also introduces a patient-centered outcome (reduced restraint use) reflecting a longer-term impact of the training, thereby improving the quality of care in the ED.

Achieving significant change with such a small percentage of staff undergoing the training shows that training even a small portion of staff in the management of agitated patients can lead to a reduction in violent patient restraint usage. As this educational session focused on the de-escalation of a violent and/or aggressive patient, data for non-violent physical restraint use was not studied for this intervention. In addition to overall reduction of restraint use noted when comparing the six-month periods before and after the intervention, there was also an overall negative trend during the post-intervention period indicating that the intervention resulted in more than a short-term improvement. As noted above, the slope of change during the six-month post-intervention was greater than the control average from the six months prior to intervention. This difference indicates a continuing trend toward lower restraint usage within the ED.

Information was gathered from a subset of nurses, protective services officers, and PSAs to assess their initial feelings about the course, their intention for future use, and relevance for clinical practice. An overwhelming majority of respondents stated that they felt more confident in safely managing agitated patients and providing a culture of respect and dignity for the patient. Over 90% of participants stated that they intended to use what they learned during the training which was born out in the decreased restraint use in the department. Nearly 90% of participants stated that the training would be beneficial to their colleagues, which helped to spur additional projects involving this curriculum. One suggested procedural change based on written feedback was to have more “roaming techs (PSAs who are assigned to float between assignments within a designated area)” in the psychiatry portion of the ED for better patient safety.

The limitations of this pilot study include the limited number of participants which creates a training disparity among members of the ED team. Furthermore, selection bias may be at play, as those staff members who frequently work in the psychiatric emergency unit may be more likely to give positive feedback to verbal de-escalation training. As a pilot study, a limited number of staff underwent the training, which could introduce confounding factors for the reduction in violent physical restraint use. Exact number of PSAs and protective service officers employed in the ED during the study period were unable to be obtained. As noted in the methods section, several staff members completed the training more than once. Another limitation involved the collection of longer-term application learning objectives. While we were able to capture the patient-centered outcome of reduced physical restraint use, no follow-up assessment was completed to evaluate participants' active use of the training. Outside of participant profession, additional demographic data related to sex, age, ethnicity, or other factors were not collected and therefore could not be stratified within the data. As an initial study, nurses, PSAs, and protective services officers were selected as participants as they often have first contact with an agitated patient. Physicians were not included in the initial portion of this study but have undergone the training in subsequent cycles after proof of concept was established. Medication use with agitated patients was not tracked within this study period. Lastly, there may have been additional changes to departmental protocols and education to which the authors were not privy which may have impacted restraint use during the 12-month period. Further studies should be conducted to determine the frequency with which de-escalation education should be offered. In addition, these studies should include not only learning outcomes, but also patient-centered and staff-centered outcomes such as length of stay and staff assaults. Lastly, as this was a pilot study, additional research should evaluate the efficacy of training larger groups of learners in verbal de-escalation.

## Conclusions

An interdisciplinary simulation-enhanced educational intervention improved learners' confidence in verbal de-escalation techniques and contributed to an overall decrease in the use of restraints. The intervention combined didactic education, focused on communication and early use of medication, with a simulated agitated patient encounter and subsequent debriefing. This educational intervention increased staff confidence in providing emotional safety for the patient, understanding of team roles during an agitated patient encounter, and ability to safely manage agitated patients.
